# Letter to the editor: Relevant errors in a systematic review and network meta-analysis evaluating the efficacy and safety of different pharmacological interventions in the treatment of tardive dyskinesia

**DOI:** 10.1007/s00228-025-03826-6

**Published:** 2025-04-15

**Authors:** Christoph U. Correll, David Rowe, Rinat Ribalov, Verena Ramirez Campos, Marco Solmi

**Affiliations:** 1 Department of Psychiatry, The Zucker Hillside Hospital, Northwell Health, Glen Oaks, NY United States; 2 Department of Psychiatry and Molecular Medicine, Donald and Barbara Zucker School of Medicine at Hofstra/Northwell, Hempstead, NY United States; 3 Department of Child and Adolescent Psychiatry, Charité – Universitätsmedizin Berlin, Berlin, Germany; 4 Medical Decision Modeling Inc, Indianapolis, IN United States; 5 Global Health Economics and Outcomes Research, Teva Pharmaceutical Industries Ltd, Tel Aviv, Israel; 6 Teva Pharmaceutical Industries Ltd., IVAX, Buenos Aires, Argentina; 7 SCIENCES Lab, Department of Psychiatry, University of Ottawa, Ottawa, ON Canada; 8 Department of Mental Health, The Ottawa Hospital, Ottawa, ON Canada; 9 Ottawa Hospital Research Institute (OHRI), Clinical Epidemiology Program University of Ottawa, Ottawa, ON Canada; 10 School of Epidemiology and Public Health, Faculty of Medicine, University of Ottawa, Ottawa, ON Canada

Ismail and coauthors [1] conducted a network meta-analysis (NMA) of randomized controlled trials (RCTs) testing treatments for tardive dyskinesia (TD) to indirectly compare and rank them. They concluded that deutetrabenazine and reserpine did not significantly outperform placebo, while vitamin E and valbenazine did. However, serious statistical, methodological, and interpretative faults and inconsistencies undermine the validity of this NMA. First, instead of calculating standardized mean differences (SMDs) with the correct formula of $SMD=\frac{mean\;difference}{{\sigma }_{pooled}}$, the authors used at least 2 incorrect formulas, such as $SMD=\frac{mean\;difference}{\sqrt{{\sigma }_{pooled}}}$ for deutetrabenazine and deanol, and $SMD=\frac{mean\;difference}{2* {\sigma }_{pooled}}$ for bromocriptine, ɣ-linolenic acid, melatonin, estrogen, and selegiline. These serious calculation mistakes led to overestimated or underestimated effect sizes, ultimately impairing the validity of the network. Second, despite stating that they included only studies using validated tools to assess TD symptoms, the authors included trials (e.g., George et al. [2]) that relied on “subjective rating” of TD. Third, the authors excluded some RCTs, such as crossover trials, because they deemed them as being at high risk of bias, but included other trials for which they underestimated the risk of bias. For instance, 5 of 6 vitamin E trials that were included in this NMA [3–7] were also analyzed in a recent Cochrane review by Soares-Weiser et al. evaluating vitamin E as a TD treatment [8]. Ismail et al. assessed all 5 as low risk of bias, while Soares-Weiser et al. assessed 4 of 5 [3–6] as having an unclear risk of bias and one [7], which showed the greatest treatment effect for vitamin E, as having a high risk of bias. Fourth, the authors excluded 2 modern, well-powered, low-risk-of-bias RCTs testing flexible doses of valbenazine and deutetrabenazine [9, 10]. This decision is based on their contradictory approaches, in which each dose of valbenazine and deutetrabenazine was assigned to a separate node while multiple doses for other interventions were grouped into a single node. Fifth, the authors did not conduct sensitivity analyses to explore sources of heterogeneity and bias at the trial level. As a matter of best practice, sensitivity analyses examining only low-risk-of-bias trials and molecules investigated in at least 2 trials would have been a minimum expectation. Sixth, Ismail et al. estimated TD treatment effects and 95% CIs but did not assess the level of certainty for these estimates as is typically done for NMAs, ideally using the Confidence in Network Meta-Analysis framework [11]. Finally, given the largely “star-shaped” and sparse network (i.e., most treatments are included in a single trial versus placebo), no meaningful and reliable ranking of interventions can be conducted, as the head-to-head comparisons are almost entirely virtual.

In summary, the inaccurate calculations and interpretation of findings resulting from the methodologically ill-informed application of powerful statistical tools, paired with inconsistent selection of evidence from existing RCTs, led the authors to misleading and potentially dangerous conclusions. Deutetrabenazine and valbenazine, two VMAT2 inhibitors for the treatment of TD, are both efficacious, as shown by high-quality, placebo-controlled RCTs [10, 12], which led to US Food and Drug Administration approval, and by a previous pairwise meta-analysis [13]. It is a fundamental error in interpretation to contradict high-quality RCT data and previous literature to conclude that a treatment is not significantly better than placebo simply because it was included in a network with high heterogeneity among other treatments. The methods, results, and conclusions of the NMA from Ismail et al. are not valid, risk to misinform guideline makers, clinicians, and persons with lived/living experience of TD, and may contribute to delayed or suboptimal care of TD. No treatment recommendations should be based on the findings of Ismail et al.

## Data Availability

No datasets were generated or analysed during the current study.
